# Intergeneric fusant development using chitinase preparation of *Rhizopus stolonifer* NCIM 880

**DOI:** 10.1186/s13568-016-0287-8

**Published:** 2016-11-14

**Authors:** Kailas D. Sonawane, Narayan R. Dandagal, Akibjaved G. Naikwadi, Piyush T. Gurav, Samar V. Anapat, Naiem H. Nadaf, Deepak B. Jadhav, Shailesh R. Waghmare

**Affiliations:** 1 Department of Microbiology, Shivaji University, Kolhapur, Maharashtra 416 004 India; 2 Department of Biochemistry, Shivaji University, Kolhapur, Maharashtra India

**Keywords:** Chitinase, *R. stolonifer*, Protoplasts, Acetone precipitation, DEAE cellulose

## Abstract

Fungal chitinase have tremendous applications in biotech industries, with this approach we focused on extracellular chitinase from *Rhizopus stolonifer* NCIM 880 for the formation of fungal protoplasts. The maximum chitinase production reached after 24 h at 2.5% colloidal chitin concentration in presence of starch as an inducer. Chitinase was extracted efficiently at 65% cold acetone concentration and then purified by using DEAE-Cellulose column chromatography. Purified chitinase having molecular weight 22 kDa with single polypeptide chain was optimally active at pH 5.0 and temperature 30 °C. The purified chitinase revealed kinetic properties like Km 1.66 mg/ml and Vmax 769 mM/min. Crude chitinase extract efficiently formed protoplasts from *A. niger, A. oryzae, T. viride* and *F. moniliforme*. The formed protoplasts of *A. niger* and *T. viride* showed 70 and 66% regeneration frequency respectively. Further, intergeneric fusants were developed successfully and identified at molecular level using RNA profiling. Thus, this study could be useful for strain improvement of various fungi for biotechnological applications.

## Introduction

Chitinase (E.C. 3.2.1.14) is a glycosyl hydrolase which catalyzes degradation of chitin polymer (Henrissat and Bairoch 1993). Chitinases have been detected in vast array of organisms including bacteria, fungi, insects, viruses, plants, and animals and may have various functions in different organisms (Mathivanan et al. 1998; Park et al. 2002; Tanabe et al. 2000; Tikhonov et al. 2002; Waghmare and Ghosh 2010a, b). Bacterial chitinases are produced to meet nutritional needs, so that chitin can be used as carbon and nitrogen sources. Plant chitinases function in self defense against pathogens having chitinous cell wall, whereas yeast and fungal chitinases are required for development and growth of the respective organisms (Gohel et al. 2006). Chitinase from *Autographa californica* nucleopolyhedrovirus was successfully expressed in transgenic tobacco plant to enhance resistance against pest and fungal pathogens (Maro et al. 2010). *Rhizopus* is a filamentous fungus and known for production of commercially important compounds such as fumaric acid and cortisone (Manosroi et al. 2006; Engel et al. 2008). Several fungi producing chitinase having diversified properties have been reviewed recently (Narayanan et al. 2014).

Protoplast fusion is an important tool to perform strain improvement as well as to manipulate and develop hybrid strains in filamentous fungi (Lalithakumari 2000). Fungal protoplasts have been used as an effective experimental biochemical tool to study cell wall synthesis, enzyme synthesis and their secretion, as well as in strain improvement for biotechnological applications. Dahiya et al. (2005) reported the effectiveness of *Enterobacter* sp. NRG4 chitinase in the generation of protoplasts from *Trichoderma reesei*, *Pleurotus florida*, *Agaricus bisporus*, and *A. niger* (Kitamato et al. 1988; Dahiya et al. 2006). Mizuno et al. (1997) isolated protoplast from *Schizophyllum commune* using the culture filtrate of *B. circulans* KA-304. An enzyme complex from *B. circulans* WL-12 with high chitinase activity was effective in generating protoplasts from *Phaffia rhodozyma* (Dahiya et al. 2005).

The present study deals with the application of crude extract obtained from the *R. stolonifer* NCIM 880 for the cost effective production of protoplasts from different fungi and to develop intergeneric fusant of *A. niger* and *T. viride*.

## Materials and methods

### Microorganisms

Cultures of *Rhizopus stolonifer* NCIM 880, *Aspergillus niger* NCIM 545 *Aspergillus oryzae* NCIM 1212 and *Fusarium moniliforme* NCIM 1099 used in this study, were obtained from National Collection for Industrial Microorganisms (NCIM), Pune, India and maintained on PDA medium.

### Screening of chitinase production

The selected *R. stolonifer* was tested for the chitinase production. The chitinase activity was screened on the colloidal chitin agar (NaNO_3_—0.3%, K_2_HPO_4_—0.1%, KCl—0.05%, MgSO_4_·7H_2_O—0.05%, FeSO_4_—0.001%, agar—2.3%, colloidal chitin—3.0%, pH 7.0) at 30 °C incubation temperature. After 48 h incubation period the plate was observed for the zone of hydrolysis around the growth.

### Production of chitinase enzyme

The fungal growth was inoculated into the flask containing 100 ml colloidal chitin medium as discussed above. The incubation was carried out at 30 °C temperature on rotary shaker for 96 h. The chitinase production was monitored by measuring chitinase activity in the cell free broth by using colloidal chitin as substrate. The methodology of the assay is described in enzyme assay section. The protein content was estimated by the Lowry method using bovine serum albumin as standard protein (Lowry et al. 1951). The effect of substrate concentration on chitinase production was studied in presence of various concentrations of colloidal chitin in the medium mentioned above, viz. 2.0, 2.5, 3.0 and 3.5%. The effect on production of chitinase was checked by addition of starch at concentration of 1% (w/v).

### Purification of chitinase

The culture of *R. stolonifer* was inoculated into the colloidal chitin containing medium and incubated for 48 h. After incubation, the supernatant was collected by centrifugation at 8000 rpm for 20 min. The obtained supernatant subjected to 30–75% ammonium sulphate precipitation and 45 to 75% cold acetone precipitation. Precipitate was collected by centrifugation at 8000 rpm for 20 min at 4 °C and dissolved in 50 mM Na-phosphate buffer having pH 7.4 and dialysed against the same buffer for overnight. Column was packed with activated DEAE-cellulose equilibrated with 50 mM sodium phosphate buffer as per earlier study (Waghmare et al. 2015). The height of column was 20 cm with the 2.5 cm diameter and protein was eluted with the 0.1–0.5 M NaCl gradient. The 50 fractions were collected having 5 ml volume of each fraction with the flow rate of 1 ml/min. All the steps were carried out at 4 °C. The collected fractions checked for the protein content by Lowry method and chitinase activity.

### Effect of pH and temperature on chitinase activity

The effect of pH on enzyme activity was studied for pH range within 3.0–10.0, using citrate-phosphate buffer (pH 3.0–5.0), sodium-phosphate buffer (pH 6.0–8.0) and glycine-NaOH buffer (pH 9.0–10.0), whereas effect of temperature on enzyme activity was studied between temperatures 10–90 °C, and chitinase activity determined as % relative activity. In the stability study of pH and temperature, enzyme was kept for 24 h at specific pH and temperature and residual activity was determined.

### Enzyme kinetics and substrate specificity

To study the *K*m and *V*max, enzyme was incubated with 0.2–2% colloidal chitin as substrate and reducing sugar was determined by DNSA method. The values of *K*m and *V*max were determined graphically using software SigmPlot version11. Substrate specificity study of chitinase was carried out using different substrate such as colloidal chitin, glycol chitin, CM-Cellulose and *p*-nitrophenyl-*N*-acetyl-β-d-glucosaminide (*p*NP-GlcNAc).

### SDS-PAGE

Purity of the fractions, showing chitinase activity, was checked by SDS-PAGE as per method discussed in Laemmli et al. (1970). The proteins were separated on 12% resolving gel and 4% stacking gel. The bands were visualized by silver staining technique (Merril 1987). The molecular weight of chitinase was determined by comparison with standard molecular marker proteins (Phosphorylase b 98 kDa, Bovine Serum Albumin 66 kDa, Ovalbumin 43 kDa, Carbonic Anhydrase 29 kDa, Soyabean Trypsin Inhibitor 20 kDa).

### Chitinase assay

Chitinase activity was quantitated by using colloidal chitin as substrate, the assay mixture includes 1 ml colloidal chitin (10 mg/ml), 0.5 ml 50 mM acetate buffer (pH 5.0) and 1 ml enzyme. The assay mixture incubated at 30 °C temperature for 1 h and activity was terminated by addition of 0.5 ml NaOH (0.5 M). The reducing sugar was determined relative to the *N*-acetyl-β-d-glucosamine standard (100–500 µg/ml concentration), by using dinitro salicylic acid (DNSA) method (Miller 1959). One unit of enzyme activity was defined as the amount of enzyme required to release 1 µmol of reducing sugar from colloidal chitin, per minute.

### Protoplasts formation

The spore suspension of *A. niger, A. oryzae, F. moniliforme* and *T. viride* was inoculated into 100 ml medium containing 20% potato infusion and 2% dextrose having pH 6.0. The flask was incubated on rotary shaker at 120 rpm for 48 h at 30 °C temperature. After incubation mycelia were separated by filtration and washed with sterile distilled water followed by 50 mM sodium phosphate buffer of pH 7.0. The washed mycelia (50 mg) were incubated with 5 ml dialysed chitinase. The protoplasts formed were examined with light microscope at 400× magnification.

### Regeneration of protoplasts

To study the regeneration ability of protoplasts, the formed individual protoplasts were spread on medium containing NaNO_3_—0.3%, K_2_HPO_4_—0.1%, MgSO_4_·7H_2_O—0.05%, FeSO_4_—0.001%, agar—2.0%, pH 7.0, with an osmotic stabilizer KCl—0.05% and sucrose 2%. Then, the plates were incubated at room temperature for 2 days. After 24 h the plates were observed for formation of mycelia under microscope. The regeneration frequency of protoplasts was measured as per the method described by Patil et al. (2015). Formation of fungal hyphae on soil media was observed under microscope at 400× magnification.

### Fusion of protoplasts

The protoplasts fusion of *A. niger* and *T. viride* was carried out by two methods i.e. method 1—self fusion and method 2—using Polyethylene glycol (PEG). In method-1, 1 ml protoplast suspension of *A. niger* and *T. viride* fungi were mixed in tube and 2 ml protoplast suspension of each kept separately in respective tubes. In method-2, same additions as per method one with additional 1 ml 30% PEG was done. Then these tubes were incubated at room temperature for 24 h. After incubation 0.1 ml aliquots from each tube was spread on Potato Dextrose Agar plates and plates were incubated at room temperature for 3–4 days. The plates were observed for fused colonies.

### Molecular characterization of fusant

The fusant obtained on the basis of colony morphology subjected to molecular characterization by using RNA profiling study. The total RNA from fusant (F1, F2, F3) and parent fungi (*A. niger, T. viride*) were extracted as per the method described by Sanchez-Rodriguez et al. (2008).

### Statistical analysis

Results obtained were the mean of three determinants and ANOVA was carried on all data at p < 0.05 using GraphPad Software.

## Results

### Screening of chitinase production of *R. stolonifer*

The mycelia of *R. stolonifer* were spread on colloidal chitin agar medium containing colloidal chitin as a sole source of carbon for the production of chitinase. After 24 h incubation period the mycelial growth was observed on the plate, but the zone of hydrolysis was observed after 48 h which confirms the ability of *R. stolonifer* to produce extracellular chitinase.

### Production of chitinase

**Fig. 1:**
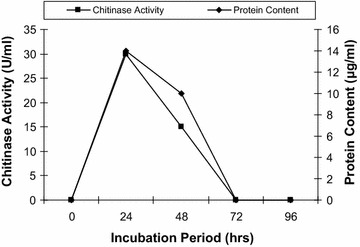
Production of chitinase by *R. stolonifer*. The fresh culture was inoculated in flask containing colloidal chitin and incubated at 30 °C then after 24 h chitinase activity and protein content was monitored

**Fig. 2:**
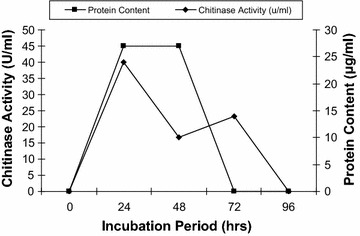
Effect of starch on production of chitinase by *R. stolonifer*. The production of chitinase was monitored in presence of starch at 30 °C

**Fig. 3:**
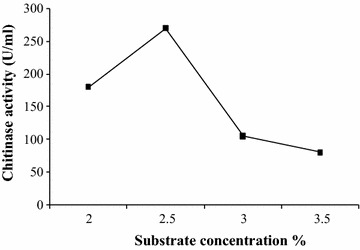
Effect of substrate concentration in medium on the production of chitinase from *R. stolonifer*. The chitinase production was studied in presence of various concentrations of colloidal chitin in the medium viz. 2.0, 2.5, 3.0, and 3.5%

The mycelial growth was inoculated into the medium containing colloidal chitin as a source of carbon and the chitinase activity was monitored with protein content. From Fig. 1, it was observed that as incubation time increases the protein content (14 µg/ml) and chitinase activity (30 U/ml) increased parallel up to 24 h, but afterwards the protein content and chitinase activity decreases drastically. It was found that *R. stolonifer* produces maximum chitinase production at 24 h incubation periods, which indicates that chitinase was produced during logarithmic phase and chitin used as sole source of carbon to obtain energy. The chitinase activity was monitored in presence of other carbon source i.e. starch. It was observed that chitinase production increased double fold in presence of starch i.e. 40 U/ml after 24 h (Fig. 2). The biomass was also found more in presence of starch. In effect of substrate concentration on the production of chitinase in the medium, the 2.5% colloidal chitin concentration was found optimum. As the substrate concentration increases more than 2.5%, the chitinase production decreases as shown in Fig. 3.

### Purification of chitinase

**Fig. 4:**
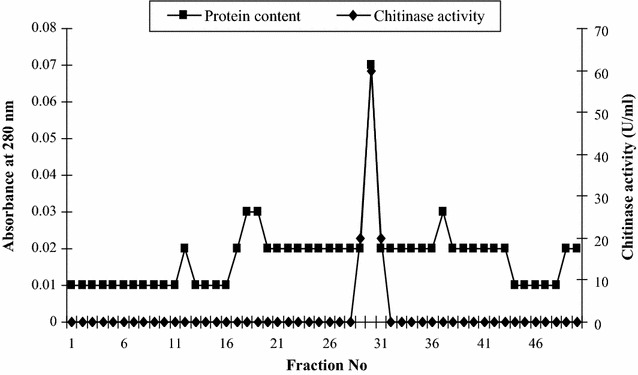
Purification profile of chitinase using ion exchange column chromatography. The chitinase precipitated by acetone method further loaded on DEAE-Cellulose column chromatography and protein was eluted with the 0.1–0.5 M NaCl gradient at the flow rate of 1 ml/min

The chitinase produced by the *R. stolonifer* is extracellular chitinase which was secreted into the medium, so it can be concentrated by the two methods i.e. fractional ammonium sulphate precipitation and organic solvent precipitation. In ammonium sulphate precipitation 50% saturated fraction showing chitinase activity, whereas in case of cold acetone precipitation 65% shows maximum chitinase activity. After acetone precipitation the extract was subjected to DEAE-cellulose ion exchange column chromatography for the purification of chitinase from *R. stolonifer.* The purification profile of chitinase is shown in Fig. 4 which reveals that the chitinase was eluted at 0.4 M NaCl.

### Characterization of purified chitinase

**Fig. 5:**
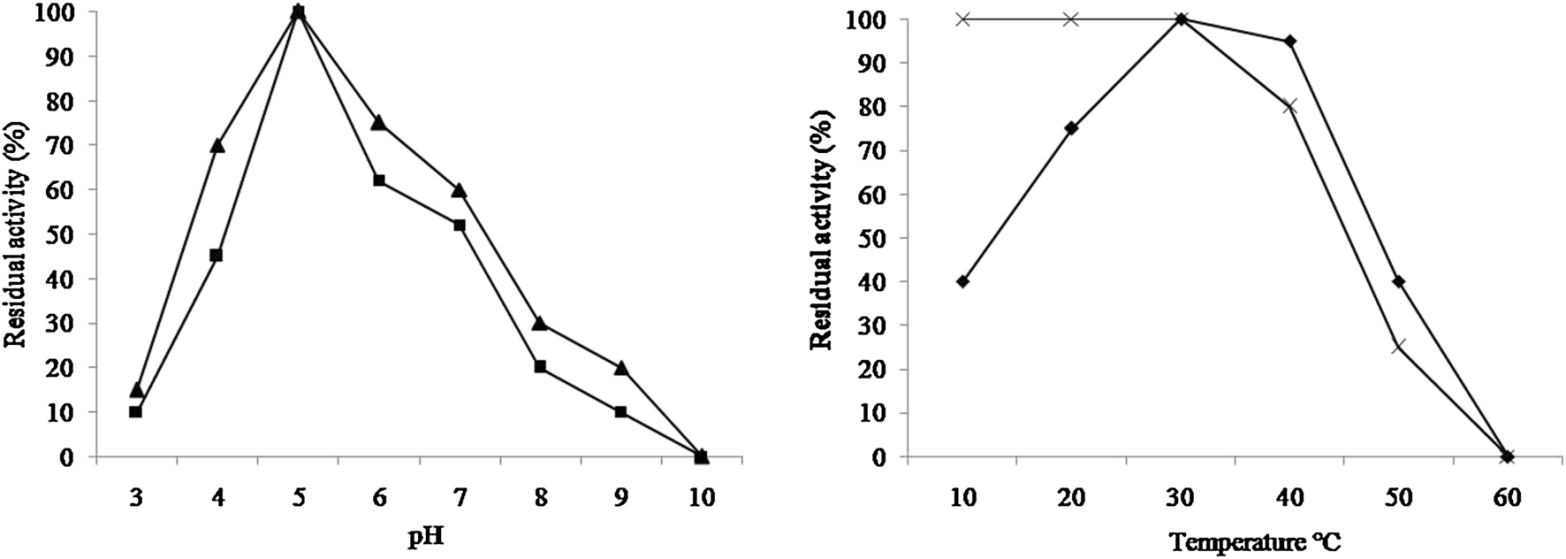
Effect of pH and temperature on chitinase activity and stability. The enzyme activity was carried out at a pH range from 3.0 to 10.0 and temperature between 10 and 60 °C. The residual activity expressed at respective pH (activity ■ and stability ▲) and Temperature (activity ■ and stability×)

**Fig. 6:**
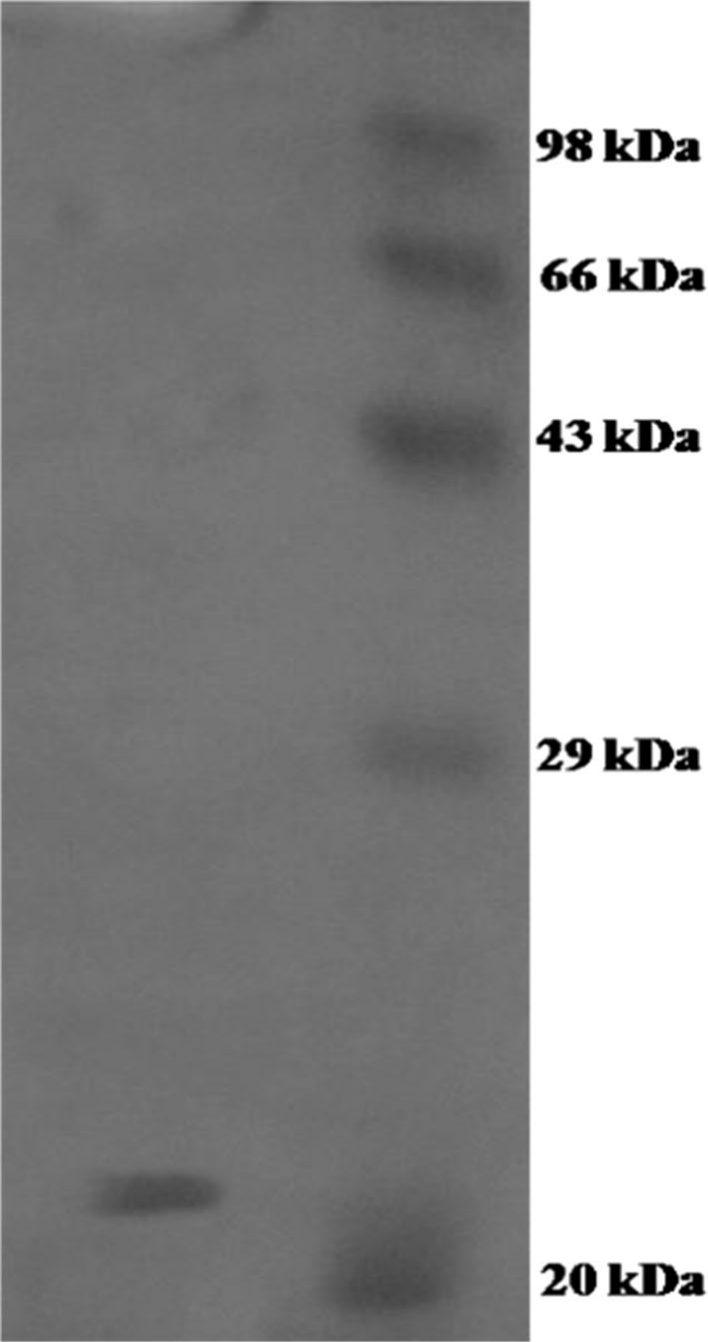
SDS-PAGE analysis of purified chitinase of *R. stolonifer*. The molecular weight of chitinase was determined by comparison with standard molecular marker proteins (Phosphorylase b 98 kDa, Bovine Serum Albumin 66 kDa, Ovalbumin 43 kDa, Carbonic Anhydrase 29 kDa, Soyabean Trypsin Inhibitor 20 kDa)

In the study of effect of pH on the chitinase activity, enzyme activity was carried out at different pH ranges from 3.0 to 10.0. The chitinase of *R. stolonifer* was found active between pH 4.0 and 7.0 and optimum at pH 5.0, as shown in Fig. 5. Enzyme stability study revealed more than 50% stability in between pH range 4 and 7. The chitinase activity was carried out at different temperature range within 10–90 °C. It was found optimum at 30 °C, but the enzyme remains active between temperature 10–50 °C and completely inactive at 60 °C (Fig. 5). Enzyme was found stable between temperatures 10–40 °C. The molecular weight of purified chitinase of *R. stolonifer* was determined by SDS-PAGE analysis. The single band of approximately 22 kDa chitinase was observed (Fig. 6).

Enzyme kinetics parameter of chitinase, *K*m and *V*max were 1.66 mg/ml and 769 mM/min respectively with colloidal chitin as substrate. The enzyme activity of *R. stolonifer* chitinase was assessed in presence of different substrates containing β-1-4 linkage, it reveals that the enzyme was highly active against colloidal chitin than the glycol chitin and *p*NP-GlcNAc (Table 1).

**Table 1:** Substrate specificity of chitinase

Substrate	Relative activity (%)
Colloidal chitin	100
Glycol chitin	82
CM-Cellulose	0
*p*NP-GlcNAc	100

### Fungal protoplast formation

**Fig. 7:**
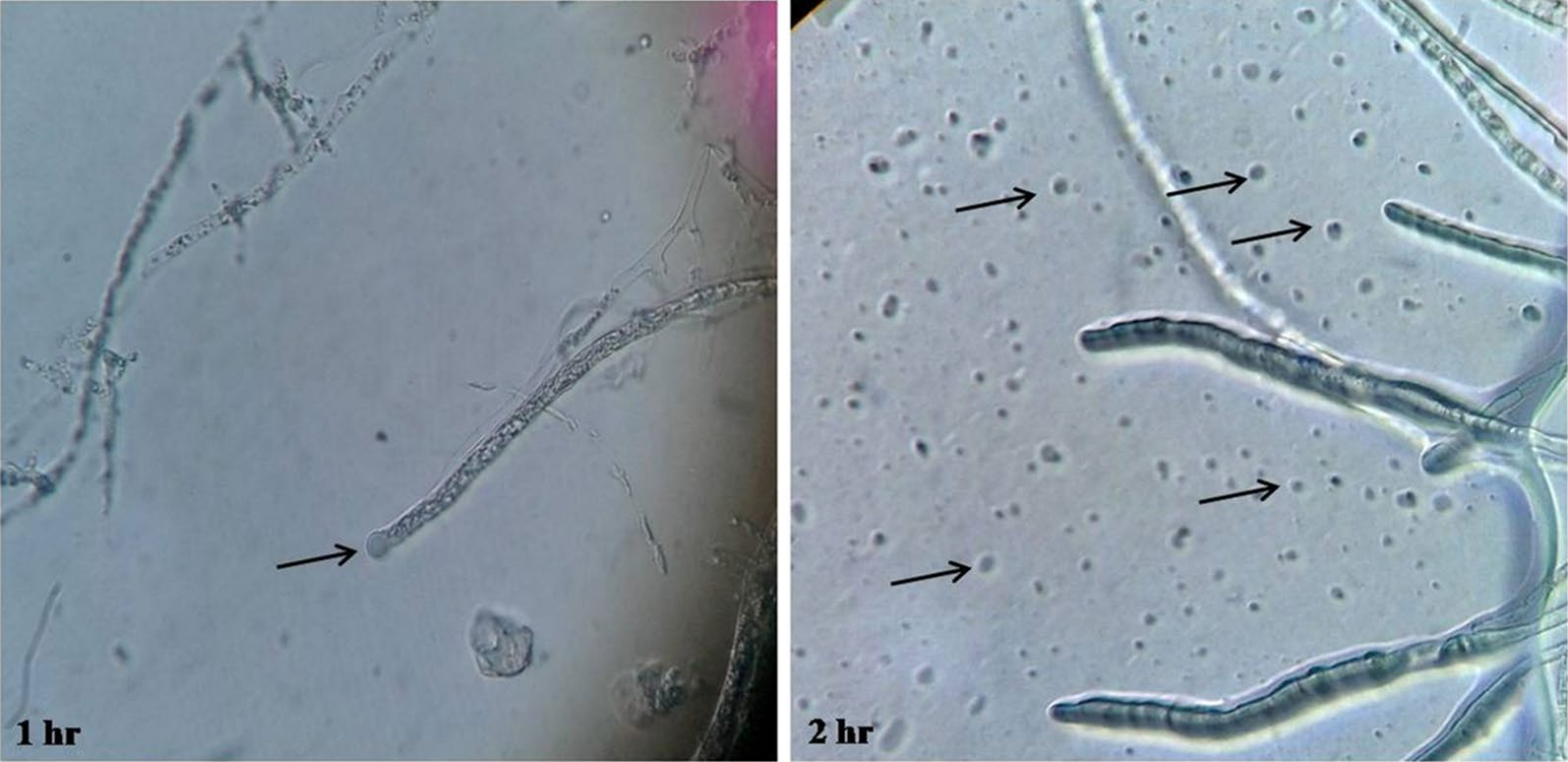
Progression of fungal protoplast after action of chitinase at different time interval. The crude chitinase was incubated with mycelium of different fungi at 30 °C and after 1 h incubation mycelia observed under microscope at ×400 magnifications

**Fig. 8:**
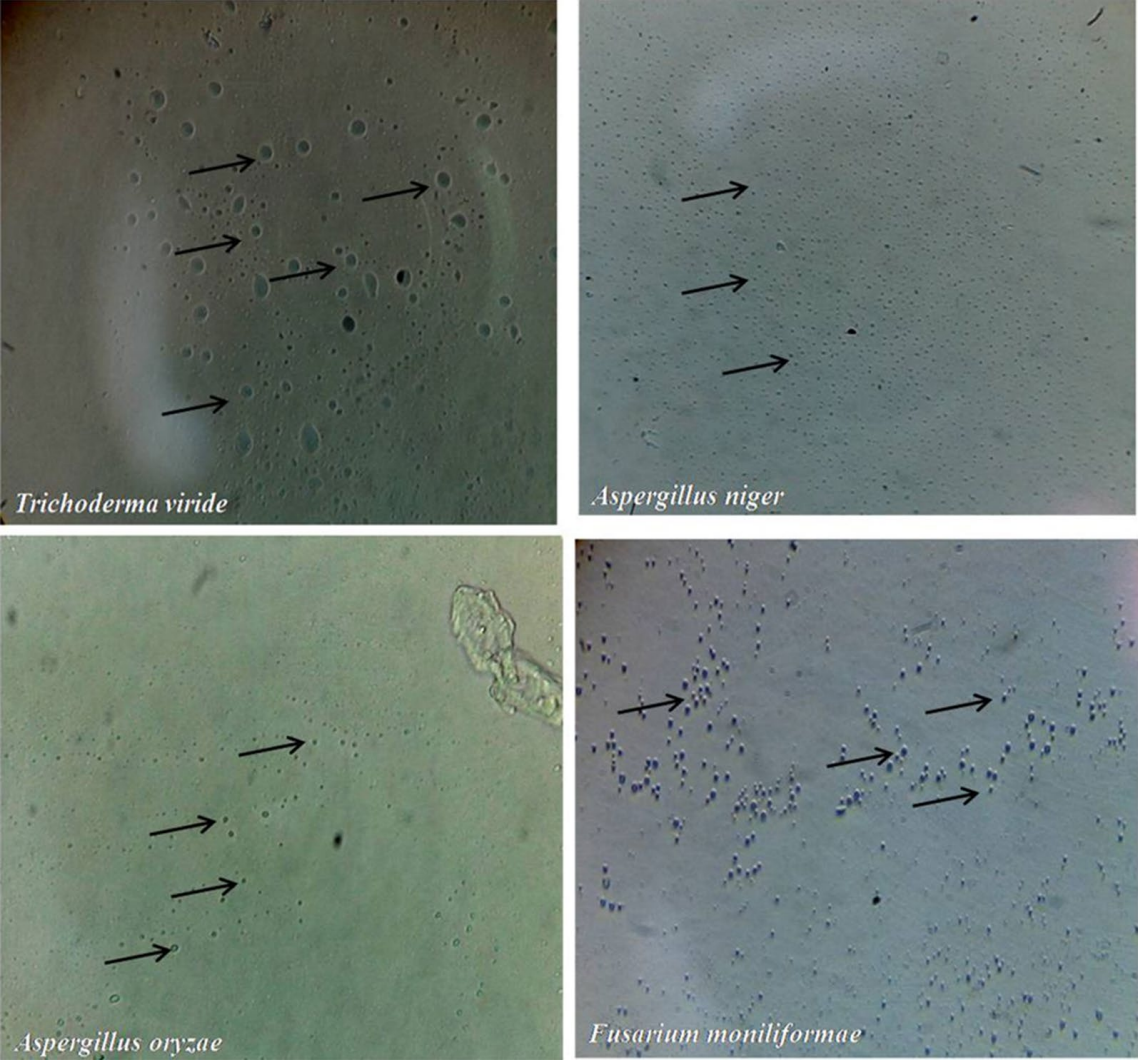
Fungal protoplast formation using chitinase of *R. stolonifer*. The mycelia of *A. niger, A. oryzae, F. moniliforme* and *T. viride* was incubated with chitinase and after 2 h of incubation formed protoplasts were observed under microscope

The chitinase which was produced by the *R. stolonifer* used for the protoplast generation of *A. niger, T. viride, F. moniliforme, and A. oryzae.* The mycelium of these fungi was incubated with chitinase for the protoplast formation. After 1 h of incubation, the mycelium of *A. niger* bulging of tip was observed and as incubation carries further the protoplast was formed (Fig. 7). The protoplasts were produced from all tested fungi such as *A. niger, T. viride, F. moniliforme, and A. oryzae* in presence of chitinase produced by *R. stolonifer* (Fig. 8).

### Protoplasts regeneration

**Fig. 9:**
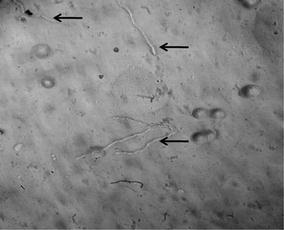
Regeneration of protoplasts on solid medium and formation of mycelium was observed under microscope at ×400 magnifications

The regeneration ability of protoplasts of *A. niger* and *T. viride* was studied on solid medium, which reveals that 70 and 66% were regenerated respectively. The regenerated protoplasts monitored under microscope, formation of budding hyphae as shown in Fig. 9. After 72 h regenerated protoplasts showed fully developed mycelium and spores.

### Protoplasts fusion

**Fig. 10:**
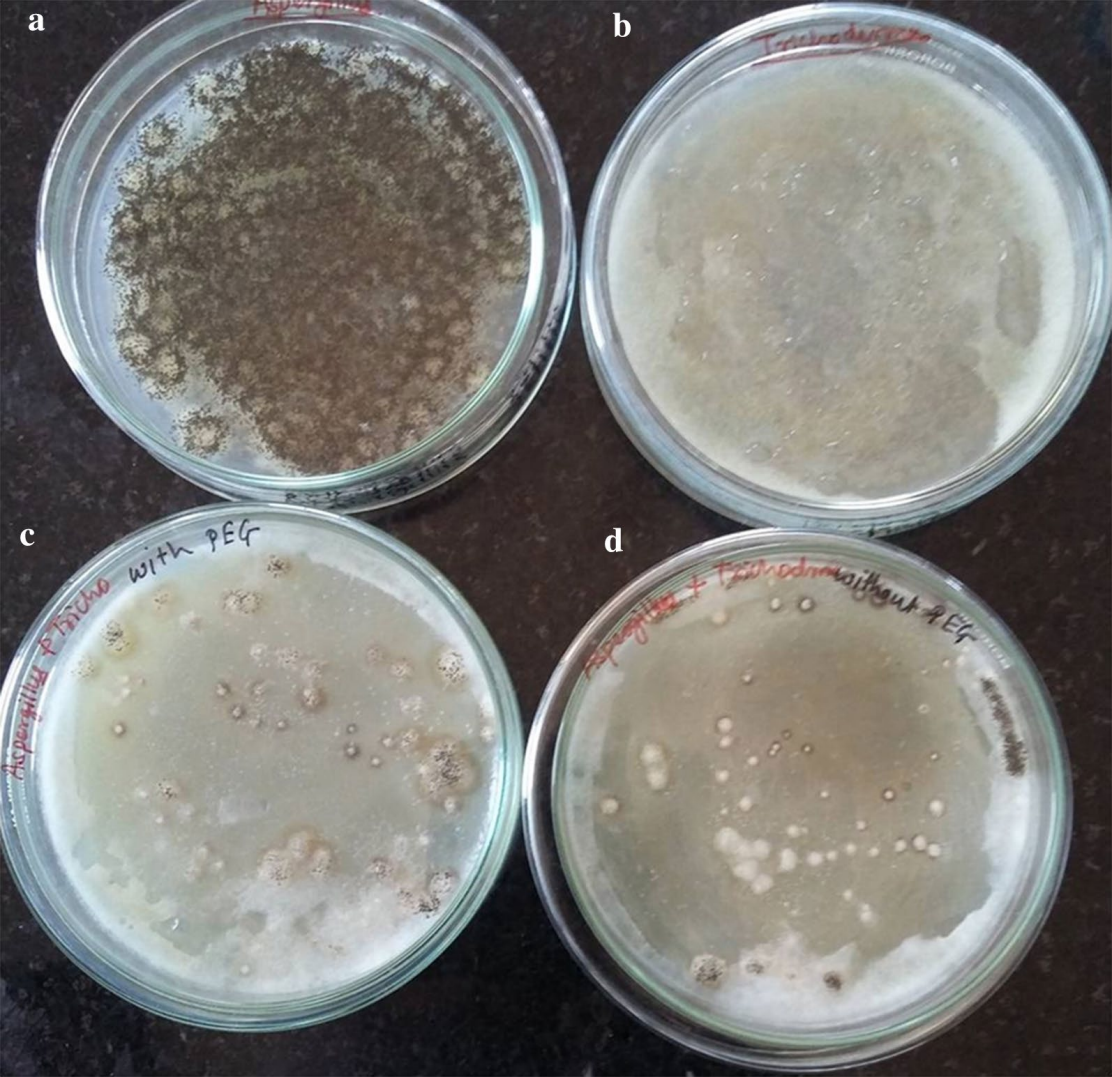
Growth of intergeneric fusant *A. niger* and *T. viride* on medium.** a** *A. niger*, **b** *T. viride*,** c** fusant formed by using PEG,** d** fusant formed by self fusion

The intergeneric protoplasts fusion was carried between *A. niger* and *T. viride* by natural self fusion and using PEG as fusion agent. Figure 10 reveals that the fusion was carried out between *A. niger* and *T. viride*, which showed different morphology of colonies compared with its parent. The more fusants were observed when the PEG used for fusion than self fusion.

### RNA profiling of fusant

**Fig. 11:**
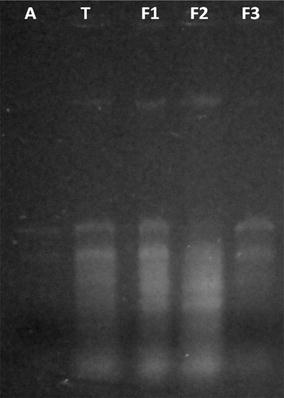
RNA profiling of parent and fused fungi. The RNA extracted from *A. niger*, *T. viride* and fusants (F1, F2, F3) were separated on 1.2% agarose gel and detected by ethidium bromide stain

The fusant obtained on the basis of differences in the formation of colonies were further analysed for expression of RNA in 24 h young mycelium. The expression studies depict that RNA expressed by fusant (F1, F2 and F3) showed different expression profile as compared to the parent (*A. niger* and *T. viride*) (Fig. 11). This confirms the successful intergeneric fusants of protoplasts obtained from *A. niger* and *T. viride* using chitinase of *R. stolonifer* NCIM 880.

## Discussion

The extracellular chitinase was extracted from fungi *R. stolonifer* NCIM 880 in presence of chitin as a substrate similar to earlier studies using *R. oligosporus* (Yanai et al. 1992) and *R*. *oryzae* (Chen et al. 2013). The fungi *R. stolonifer* NCIM 880 produces maximum chitinase at 2.5% colloidal chitin concentration in the medium comparatively higher than the reported 1% of *Penicillium* sp. LYG 0704 (Lee et al. 2009), 1% of *A. carneus* (Abde-Naby et al. 1992), 2% of *P. ochrochloron* MTCC 517 (Patil et al. 2013). Increase in chitinase production along with fungal biomass was observed in presence of starch, which suggests that *R. stolonifer* utilizes starch more rapidly than the colloidal chitin.

The extracellular chitinase produced by *R. stolonifer* was extracted by Ammonium sulphate and cold acetone precipitation method. Among these two methods cold acetone precipitation method was found more suitable for precipitation which gives 12 fold more yield than the ammonium sulphate precipitation. Similarly Lee et al. (2009) reported precipitation of chitinase produced from *Penicillium* sp. LYG 0704 by using isopropanol (Lee et al. 2009). The chitinase was purified by one step purification technique using DEAE-Cellulose ion exchange column chromatography. The eluted single peak showing chitinase activity indicates that *R. stolonifer* produces single enzyme which hydrolyzes chitin efficiently, where as *R. oligosporus* produces two chitinases (Yanai et al. 1992). The purified enzyme was optimally active at pH 5.0 and temperature 30 °C which was similar to that of chitinase produced by various fungi such as *Fusarium chlamydosporum* (Mathivanan et al. 1998), *Metarhizium anisopliae* (Kang et al. 1999), *Penicillium* sp. LYG0704 (Lee et al. 2009). Chitinase purified by ion exchange chromatography of *R. stolonifer* shows single polypeptide chain with low molecular weight as confirmed by SDS-PAGE. The approximate molecular weight of chitinase produced by *R. stolonifer* was 22 kDa, which is comparatively lower than the 50 kDa of *R. oryzae* (Chen et al. 2013), 68 kDa of *P. ochrochloron* MTCC 517 (Patil et al. 2013). *K*m of *R. stolonifer* chitinase was 1.66 mg/ml which was lower as compared to other chitinases like 4.02 mg/ml of *Rhizomucor miehei* (Yang et al. 2016), but higher than the 0.82 mg/ml chitinase of *Lecanicillium lecanii* (Nguyen et al. 2015).

Protoplast have been formed from the various genera of fungi by using mixtures of different enzymatic preparations including β,1-3 glucanase, chitinase and protease (Robinson and Deacon 2001; Solis et al. 1996; Stasz et al. 1988; Tilburn et al. 1983; Waghmare et al. 2011). In this study we have used crude extract of chitinase produced from *R. stolonifer* to form protoplasts from various fungi such as *A. niger, A. oryzae, T. viride, F. moniliforme.* The protoplasts were released efficiently from all these tested fungi alone by using chitinase of *R. stolonifer*, compared with protoplasts generated by using enzymatic preparations of *T. harzianum* contains β,1-3 glucanase and chitinase (Yanai et al. 1992). So these results indicate that chitinase of *R. stolonifer* has a broad activity for protoplasts formation among various genera of fungi.

The regeneration of protoplasts into mycelium is an important aspect to study the stability and gene expression of fungi. Protoplasts of *A. niger* formed by using chitinase preparation of *R. stolonifer* shown 70% regeneration frequency in medium containing osmotic stabilizer KCl- 0.05% and sucrose 2% at pH 7.0, which was higher than the earlier report (Bekker et al. 2009). The intergeneric fusion between *A. niger* and *T. viride* was achieved using 30% PEG and fusants were observed on the basis of phenetic and molecular characterization. *In*-*vivo* RNA profiling study has been found convenient for the identification of fusants. Earlier, Santos et al. (1994) reported identification of pathogenic *Candida* species on the basis of transfer RNA profiling. Similarly, Zhou et al. (2012) discussed genome wide identification on profiling of microRNA-like RNAs from *Metarhizium anisopliae* during development. Likewise, Patil et al. (2015) reported intergeneric fusion between *A. oryzae* and *T. harzianum*. Strom and Bushley (2016) described role of heterokaryotic fungi with distinct traits for antibiotic and enzyme production, fermentation, biocontrol, and bioremediation.

Here, we first time report the production of extracellular chitinase from *R. stolonifer* NCIM 880. Crude chitinase preparation has ability to form protoplasts of various genera of fungi with high regeneration frequency rate. The successfully formed intergeneric fusant of *A. niger* and *T. viride* have been identified using RNA profiling. Thus, the formed heterokaryotic fungi would be useful in various sectors of industries for biotechnological applications.
